# Editorial: The Response of Mucosal Epithelial Cells to Infections

**DOI:** 10.3389/fcimb.2020.602312

**Published:** 2020-11-16

**Authors:** Ran Wang, David Proud

**Affiliations:** ^1^ Mater Research Institute, The University of Queensland, Brisbane, QLD, Australia; ^2^ Cumming School of Medicine, University of Calgary, Calgary, AB, Canada

**Keywords:** infections, antigen sensing and presentation, innate immune cells, cytokines, chemokines, mucosal epithelial cells, pathogens, host defense

Mucosal epithelial cells of the respiratory, gastrointestinal, and genital tracts are the initial site of interaction with pathogens. Although initially viewed primarily as functioning as physical barriers to infection, the past several decades have shown a far more complex set of interactions. Pathogens can interact directly with mucosal epithelia, either *via* specific receptors, or with any of several families of pattern recognition receptors (PRR). These interactions can trigger a wide range of cellular functions, including altering cellular metabolism, regulation of barrier function and the generation of innate immune responses designed to limit infections. The cellular consequences of infection can, however, also exacerbate disease states. The current special issue of the journal covers a number of specific aspects of the interactions of infectious agents with epithelial cells at mucosal surfaces. The papers include insightful review articles as well as a number of original investigations.

Six out of the eight manuscripts included in this Research Topic examine the role of airway epithelial cells in the response to inhaled pathogens. Human rhinoviruses (HRV) infections are a major trigger of acute exacerbations of asthma, chronic obstructive pulmonary disease, and cystic fibrosis (Leigh and Proud, 2015). The review by Ganjian et al. provides an overview of rhinoviruses and how they are recognized by human airway epithelial cells (HAE). This includes recognition of HRV replication intermediates by PRR and the proinflammatory and antiviral responses triggered by such interactions. The review also considers various signaling pathways that regulate epithelial responses to HRV infections and discusses differences in responses between cells from normal subjects and those with lower airway diseases, including potential differences in antiviral responses between patient populations.

Jamieson et al. focus on the role of epithelial IL-17C, a novel member of the IL-17 family of cytokines, in response to HRV infection. Using highly differentiated HAE they demonstrate that HRV infection triggers production of IL-17C exclusively in the basolateral direction. They further show that basolateral, but not apical, stimulation of HAE with IL-17C led to basolateral release of the neutrophil chemoattractant CXCL1. This suggests that basolateral release of IL-17C during HRV infections can feedback to induce basolateral release of CXCL1 leading to neutrophil recruitment. Since neutrophils correlate with disease severity, IL-17C may be a contributor to increased inflammation and symptoms during infections.

An important component of acute exacerbations of lower airway diseases is mucus hypersecretion. Wang et al. showed that infection of differentiated HAE with either of two different strains of HRV increased gene expression of the mucins MUC5AC and MUC5B and caused increased secretion of mucin proteins. They further show that HRV-induced mucin expression appears to be regulated by the transcription factor SAM-pointed domain-containing Ets-like factor (SPDEF) and SPDEF-regulated genes, as well as *via* purinergic receptor signaling in response to extracellular ATP. Finally, they showed that the muscarinic receptor antagonist, tiotropium bromide, as well as the glucocorticoid, fluticasone propionate, inhibit HRV induced mucin expression without affecting viral loads. These effects may underlie the beneficial effects of these drugs in treating exacerbation of lower airway diseases.

It has previously been shown that knockdown of the E3-ubiquitin ligase, Pellino-1, reduces the inflammatory response in sepsis (Chang et al., 2009). Marsh et al. show here that Pellino-1 is expressed in the airway epithelium and plays a role in regulating responses to viral infection. Although knockdown of Pellino-1 in primary bronchial epithelial cells led to reduced production of proinflammatory cytokines in response to the synthetic double-stranded RNA (dsRNA) mimic, Poly I:C, the authors found that Pellino-1 knockout mice actually showed enhanced inflammatory cytokine production upon infection with viruses.

Cao et al. focused on the role of a specific ATP binding cassette (ABC) transporter, ABCF1, in epithelial responses to dsRNA mimics. This ABC transporter has been identified as a cytosolic nucleic acid sensor. Here it shown that ABCF1 is expressed in epithelial cells and mediates the production of CXCL10 by dsRNA mimics. Knockdown experiments and gene ontogeny analysis suggest that, while ABCF1 does play a role in regulation of antiviral responses, it is most strongly associated with Toll-like receptor signaling, suggesting a multifactorial role in epithelial innate immunity.

Airway bacterial infections can also cause substantial morbidity and mortality. Ahn and Prince review the role of IL-22 and IFN-λ in regulating epithelial responses to bacterial infection. These two cytokines both signal through the cognate receptor IL-10RB and have been linked to regulation of epithelial barrier function. IL-22 induces production of antimicrobial peptides and plays a role in increasing junctional proteins. By contrast, IFN-λ has known antiviral and proinflammatory proteins and reduces junctional integrity, which would be expected to facilitate bacterial and immune cell translocation. Because the actions of these cytokines can vary depending on the nature of the pathogen and the specific tissue microenvironment, additional studies are needed to fully understand the role of these cytokines in airway defense and to identify targets that may have therapeutic potential.

Cell surface mucin MUC1, which is highly expressed on gastrointestinal epithelial cells, is known to limit *Helicobacter pylori (H. pylori)* infection in humans and mice (Linden et al., 2009). Sheng et al. investigated the molecular network modulated by MUC1 in the gastric mucosa of *H. pylori* infected mice using microarray. Over the 72 h of infection, genes regulating lipid metabolism are firstly suppressed then promoted in *Muc1^−/−^* compared to *WT* control, supporting the role of MUC1 in driving metabolic changes in epithelial cancers. As infection progresses, a network of anti-inflammatory genes is upregulated, which correlates with the increased bacteria load in *Muc1^−/−^* compared to *WT* control. Since MUC1 is expressed in both epithelial and immune cells in the gastric mucosa, further studies are needed to dissect the epithelial vs immune contribution in the temporal regulation of MUC1 during *H. pylori* infection.

Human papillomavirus (HPV) infections are responsible for 99% of cervical cancer (Burd, 2003). Budhwani et al. review the underlying causes for HPV infection associated cancer development in a specific region of cervix—squamo-columnar junction (SCJ). HPV replication y modulates genes involved in stem-cell maintenance in cervical mucosal SCJ, which further leads to HPV-mediated cervical malignancy. Collective evidence highlights SCJ mucosa being a vulnerable site for HPV infection due to its unique anatomical location, altered host-defensin production, lack of immune cells and presence of cancer stem cell niche.

## Author Contributions

RW and DP wrote the editorial together. All authors contributed to the article and approved the submitted version.

## Conflict of Interest

The authors declare that the research was conducted in the absence of any commercial or financial relationships that could be construed as a potential conflict of interest.

## References

[B1] Burd E. M. (2003). Human papillomavirus and cervical cancer. Clin. Microbiol. Rev. 16 (1), 1–17. 10.1128/CMR.16.1.1-17.2003 12525422 PMC145302

[B2] Chang M. Jin W. Sun S. C. (2009). Peli1 facilitates TRIF-dependent Toll-like receptor signaling and proinflammatory cytokine production. Nat. Immunol. 10 (10), 1089–1095. 10.1038/ni.1777 19734906 PMC2748822

[B3] Leigh R. Proud D. (2015). Virus-induced modulation of lower airway diseases: pathogenesis and pharmacologic approaches to treatment. Pharmacol. Ther. 148, 185–198. 10.1016/j.pharmthera.2014.12.005 25550230 PMC7173263

[B4] Linden S. K. Sheng Y. H. Every A. L. Miles K. M. Skoog E. C. Florin T. H. J. (2009). MUC1 Limits Helicobacter pylori Infection both by Steric Hindrance and by Acting as a Releasable Decoy. PloS Pathog. 5 (10), e1000617. 10.1371/journal.ppat.1000617 19816567 PMC2752161

